# NGS Analysis Confirms Common *TP53* and *RB1* Mutations, and Suggests *MYC* Amplification in Ocular Adnexal Sebaceous Carcinomas

**DOI:** 10.3390/ijms22168454

**Published:** 2021-08-06

**Authors:** Cornelia Peterson, Robert Moore, Jessica L. Hicks, Laura A. Morsberger, Angelo M. De Marzo, Ying Zou, Charles G. Eberhart, Ashley A. Campbell

**Affiliations:** 1 Department of Molecular and Comparative Pathobiology, The Johns Hopkins University School of Medicine, Baltimore, MD 21205, USA; cpeter52@jhmi.edu; 2 Department of Pathology, The Johns Hopkins University School of Medicine, Baltimore, MD 21205, USA; robert.f.moore@gmail.com (R.M.); jbeckman@jhmi.edu (J.L.H.); lmorsber@jhmi.edu (L.A.M.); ademarz@jhmi.edu (A.M.D.M.); yzou19@jhmi.edu (Y.Z.); 3 Clinical Cytogenetics Laboratory, The Johns Hopkins University School of Medicine, Baltimore, MD 21205, USA; 4 Johns Hopkins Genomics, The Johns Hopkins University School of Medicine, Baltimore, MD 21205, USA; 5 Sidney Kimmel Comprehensive Cancer Center, The Johns Hopkins University School of Medicine, Baltimore, MD 21205, USA; 6 The Brady Urological Research Institute, The Johns Hopkins University School of Medicine, Baltimore, MD 21205, USA; 7 Department of Ophthalmology, The Johns Hopkins University School of Medicine, Baltimore, MD 21205, USA

**Keywords:** sebaceous carcinoma, ocular adnexal tumor, next-generation sequencing, MYC

## Abstract

Ocular adnexal (OA) sebaceous carcinomas generally demonstrate more aggressive clinical and histopathological phenotypes than extraocular cases, but the molecular drivers implicated in their oncogenesis remain poorly defined. A retrospective review of surgical and ocular pathology archives identified eleven primary resection specimens of OA sebaceous carcinomas with adequate tissue for molecular analysis; two extraocular cases were also examined. Next-generation sequencing was used to evaluate mutations and copy number changes in a large panel of cancer-associated genes. Fluorescence in situ hybridization (FISH) confirmed *MYC* copy number gain in select cases, and immunohistochemistry to evaluate MYC protein expression. The commonest mutations occurred in *TP53* (10/13) and *RB1* (7/13). Additional mutations in clinically actionable genes, or mutations with a frequency of at least 25%, included the *NF1* (3/12), *PMS2* (4/12), *ROS1* (3/12), *KMT2C* (4/12), *MNX1* (6/12), *NOTCH1* (4/12), *PCLO* (3/12), and *PTPRT* (3/12) loci. Low level copy number gain suggestive of amplification of the *MYC* locus was seen in two cases, and confirmed using FISH. MYC protein expression, as assessed by immunohistochemistry, was present in almost all sebaceous carcinoma cases. Our findings support the concept that alterations in *TP53* and *RB1* are the commonest alterations in sebaceous carcinoma, and suggest that *MYC* may contribute to the oncogenesis of these tumors.

## 1. Introduction

Nearly 40% of sebaceous carcinomas arise from the sebaceous glands of the periocular region, where they represent approximately 5% of all epithelial eyelid malignancies [1,2,3,4]. These ocular adnexal (OA) sebaceous carcinomas can have variable presentations, including cases resembling benign inflammatory lesions, such as blepharoconjunctivitis or chalazion, or other types of malignancy, often leading to a delay in diagnosis and surgical intervention [3,5,6]. Sebaceous carcinomas in this region are relatively aggressive, and tumor recurrence has been demonstrated in 18% of cases, metastasis in 7–21%, and mortality in 6–20% [4,7,8]. Intraepithelial or pagetoid spread, particularly in the conjunctiva, occurs in approximately 50% of OA sebaceous carcinomas, and is thought to contribute to a more aggressive phenotype than that seen in extraocular tumors [7].

A number of mutations have been identified in sebaceous carcinoma, but molecular drivers remain incompletely understood. The low incidence of 0.16/100,000 person-years for sebaceous carcinoma in the US, as well as the small size of many biopsy specimens, means that it is difficult for a single institution to accumulate large cohorts with excess tissue for molecular analysis. [9] However, several studies have identified mutations in tumor suppressor genes, including *TP53* and *RB1*, and p16 expression has been implicated as a robust immunohistochemical marker for these tumors [10,11,12,13,14,15,16,17,18,19]. High-risk human papillomavirus (HPV), whose oncogenes encode well-characterized inhibitors of p53 and Rb, has been detected in up to 18% of OA sebaceous carcinomas without concurrent tumor suppressor mutations, and additional studies have demonstrated overexpression of miRNAs that influence the p53 suppressor complex [6,11,20,21,22]. 

Sebaceous proliferations, most commonly benign sebaceous adenomas or sebaceomas, but also sebaceous carcinomas, can occur in association with visceral malignancies in Muir-Torre Syndrome (MTS), a phenotypic subset of hereditary non-polyposis colon cancer syndrome (HNPCC), or Lynch syndrome [7,23]. Affected patients demonstrate microsatellite instability (MSI) and autosomal dominant loss-of-function mutations in genes encoding DNA mismatch repair (MMR) proteins, including MLH1, MSH2, and less frequently, MSH6, PMS1, and PMS2 [7,24,25,26,27,28,29,30,31,32]. However, loss of MMR protein expression and/or MSI occurs more commonly in extraocular than OA sebaceous carcinomas [7,33].

While mutations involving tumor suppressor complexes and DNA repair mechanisms have been documented, further elucidation of the molecular drivers in sebaceous carcinoma oncogenesis could provide a more targeted treatment approach to minimize the need for aggressive surgical resection, currently challenged by tumor multicentricity, skip lesions, and pernicious intraepithelial spread. The objectives of the current study were to identify molecular drivers of OA sebaceous carcinoma utilizing Next-generation sequencing (NGS), and to validate novel alterations using appropriate techniques. 

## 2. Results

### 2.1. Clinicopathologic Characteristics

Eleven primary sebaceous carcinoma specimens from OA sites with enough tissue for NGS analysis were identified from retrospective review, along with 2 from extraocular sites. In this cohort, tumors arose in 6 males and 7 females, all of whom were Caucasian. The mean age of patients at clinical presentation was 72 years (range 43–90). Seven patients reported a history of prior malignancy, and 1 had a diagnosis of Muir-Torre syndrome. OA tumors were predominantly localized to the upper lid (*n* = 9), but were also identified in the lower lid (*n* = 2). None of the tumors were associated with local or distant metastases. 

Histological features of the tumors included combined intraepithelial and subepithelial involvement (*n* = 6), intraepithelial involvement alone (*n* = 1), and subepithelial involvement alone (*n* = 2). Based on the AJCC 8th edition TMN classification, 11 cases were classified as Tis/T1 and 2 as T2. The mean size of the tumors was 7.5 mm. Clinicopathologic characteristics for each case are shown in Table 1.

### 2.2. Mutations in Sebaceous Carcinoma

The NGS panel used detects mutations in 435 cancer-related genes, and for 64 of these loci, copy number variations (CNV) can also be identified. Due to limited DNA, a smaller 27 gene NGS panel with no copy number analysis was performed in Case11. Two hundred and forty-five mutations were identified in 205 genes in the 13 sebaceous carcinomas (2–46 genes mutated per tumor, mean 15.9 ± 12.9). All sebaceous carcinomas evaluated harbored at least 1 mutation in a clinically actionable gene, or in genes affecting pathways for which known therapeutic agents are available [34]. The mean number of genes with clinically actionable mutations in OA tumors was 4.4 ± 3.4 (range 1–13), while in extraocular tumors, the mean number of clinically actionable alterations was 5.5 ± 0.7 (range 5–6).

The most frequently encountered genetic alterations occurred in *TP53* (10 mutations in 13 tumors, 76.9%) and *RB1* (7 mutations in 13 tumors, 53.8%). Interestingly, while 10 out of 11 OA tumors (90.9%) harbored *TP53* mutations, neither of the 2 extraocular tumors demonstrated a mutation in this gene. All tumors (7/7) that harbored an *RB1* mutation had *TP53* computations, while 3/10 (30%) of OA tumors harboring *TP53* mutations were wild-type for *RB1*. Six of the 10 *TP53* alterations were identified as missense mutations, 2 were nonsense mutations, and the remaining 2 were intronic substitutions. One of the 7 *RB1* alterations (14.3%) in the OA tumors was a deletion, 2/7 (28.6%) were nonsense mutations, 1/7 (14.3%) was a missense mutation, and 3/7 (42.9%) were intronic substitutions (see Table 2).

Additional clinically actionable alterations were noted in *ABL1*, *FGFR2*, *IDH2*, *MLH1*, *PDGFRA*, *PTEN*, *RET*, *SMO*, *TSC1*, and *TSC2* in 1/13 tumors (7.7%), *BRCA2*, *MSH2*, *MSH6*, *MTOR*, *NTRK1*, *POLD1*, *PTCH2*, and *TERT* in 1/12 tumors (8.3%), *EGFR*, *FGFR3*, *HRAS*, and *PIK3CA* in 2/13 tumors (15%), *ATM*, *BRCA1*, *NTRK3*, and *PALB2* in 2/12 tumors (16.7%), NF1 and ROS1 in 3/12 tumors (25%), and PMS2 in 4/12 tumors (33.3%). 

Other genes with an alteration frequency of at least 3/12 (25%) included *KMT2C* in 4/12 (33%), *MNX1* in 6/12 (50%), *NOTCH1* in 4/12 (33%), *PCLO* in 3/12 (25%), and *PTPRT* in 3/12 (25%). There were no clear differences in the frequency of these changes between OA and extraocular tumors. Both *NOTCH1* mutations in OA tumors and 1/2 mutations in extraocular tumors were indels, while the remaining extraocular *NOTCH1* alteration detected was a missense mutation. Mutations in *KMT2C* were missense mutations in 2/2 OA tumors and deletions in 2/2 extraocular tumors. None of these genes could be evaluated in Case 11, as they are not included in the limited NGS panel used.

### 2.3. Copy Number Variations in Sebaceous Carcinoma

Copy number gains (mean log_2_ ratio over 0.5, but less than 1.3) were identified in 33 genes across 11 tumors (range: 3–14, mean 6.6 ± 3.5 CNVs/tumor). Only one loss was identified among 11 tumors, which included the *AR* locus. CNVs with a frequency of ≥18% included equivocal copy number gains in *BAP1*, *CCND3*, *CCNE1*, *EZH2*, *MDM2*, *PIK3CA*, and *PMS2* each in 2/11 tumors (18.2%), *CDK4*, *KRAS, PTEN*, and *XPO1* each in 3/11 tumors (27.3%), *AURKA* and *CDKN2A* each in 4/11 tumors (36.4%), and *CDKN2B* and *MYC* each in 6/11 tumors (54.5%) (see Supplemental Figure S1). Low level copy number gains suggestive of amplification (mean log_2_ ratio of 1.3 or greater) occurred only in *MYC* in 2/11 tumors (18.2%), both of which were OA. There were no significant differences between patient sex, age at presentation, tumor location, or laterality with respect to *MYC* CNV (see Table 3). 

### 2.4. MYC Fluorescence In Situ Hybridization (FISH)

**Figure 1:**
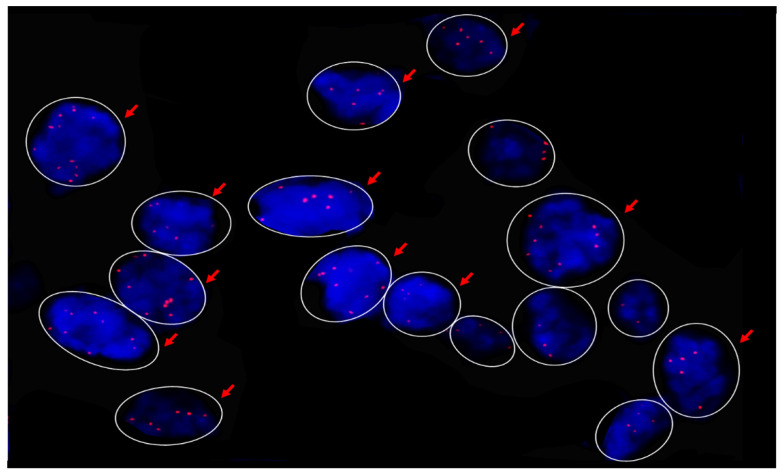
FISH detecting signals within the 8q24.21 locus in a sebaceous carcinoma from Case 8. Arrows indicate nuclei with five or more MYC signals.

FISH analysis using probes that map to 8q24 confirmed increased MYC copy number in the two OA tumors with amplification suggested by NGS. Case 8 had MYC amplification in 76% of cells analyzed, with 55% of nuclei demonstrating five or more signals (see Figure 1). Case 9 had MYC amplification in 70% of cells analyzed, with 58% of nuclei demonstrating five or more signals.

### 2.5. MYC Immunohistochemistry

**Figure 2:**
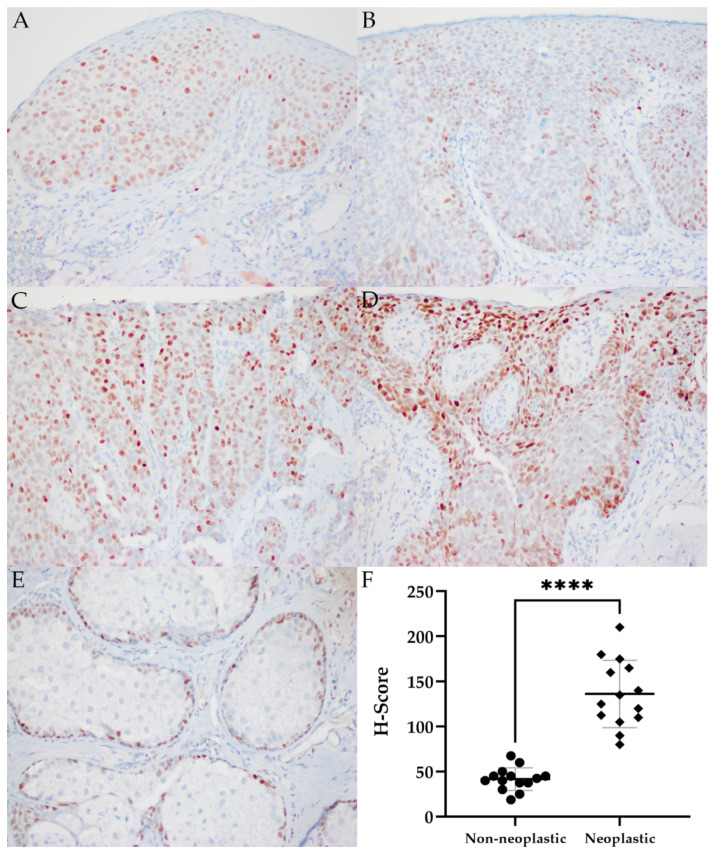
MYC immunohistochemistry of intraepithelial sebaceous carcinomas without *MYC* copy gain in Case 10 (**A**) and Case 3 (**B**) and with *MYC* copy gain in Case 4 (**C**) and Case 6 (**D**). Representative image of non-neoplastic sebaceous glands with MYC immunolabeling of basal cells (**E**). H scores for neoplastic sebaceous glands were significantly greater than those for non-neoplastic sebaceous glands. Data are shown as mean ± SD (**** *p* ≤ 0.0001) (**F**).

We next examined the expression of MYC protein in all sebaceous carcinomas that were subject to NGS, as well as four additional OA tumors, using immunohistochemistry. Sebaceous carcinomas with and without MYC copy gain demonstrating a range of expression are shown in Figure 2. Neoplastic sebaceous glands (136.3 ± 37.4) had significantly greater h scores (*p* ≤ 0.001) compared to adjacent normal glands (41.7 ± 12.7). High levels of protein expression (MYC h scores ≥ 100) tended to occur more commonly in OA tumors than extraocular tumors. There was no correlation between MYC copy gain and MYC immunolabeling (*p* = 1.00), tumor size (*p* = 0.50) or concomitant mutations in TP53 or RB1 (*p* = 1.00) (see Table 4).

## 3. Discussion

Somatic *TP53* and *RB1* mutations are among the most frequently reported in sebaceous carcinomas. In this cohort, 77% and 54% of all sebaceous carcinomas harbored these mutations, respectively. *TP53* mutations were only identified in OA tumors, and the frequency in our group was similar to that reported in prior studies [11,24,35]. One previous study reported aberrant immunolabeling, potentially corresponding to *TP53* mutations in 19/29 (66%) of OA sebaceous carcinomas; in another series, *TP53* mutations were detected in 23/31 (71%) of cases [11,35]. Additionally, overexpression of Has-miR-34a, part of the TP53 suppressor complex, has been reported in both nodular and pagetoid sebaceous carcinomas, and nuclear expression has been observed in 68% of intraepithelial tumor cells in OA cases [10,22]. *RB1* mutations were reported in 14/29 (48%) and 12/31 (39%) of OA tumors in two studies [11,35].

*NOTCH1* alterations were present in 33% of all sebaceous carcinomas in the present study, with indels observed in 2/2 of OA and 1/2 extraocular tumors harboring a mutation. *NOTCH* family mutations have been reported in OA metastases and primary extraocular sebaceous carcinomas in one study, and 8/31 (26%) of tumors harbored *NOTCH1* mutations in another [24,35]. In both of these series, *NOTCH1* mutations occurred concomitantly with *TP53* and *RB1* mutations in the majority of cases [24,35]. Here, 2/2 of the OA tumors with *NOTCH1* mutations were also *TP53*/*RB1* double mutants, while neither of the extraocular tumors with *NOTCH1* mutations had either *TP53* or *RB1* mutations. Interestingly, one study concluded that OA sebaceous carcinomas with *TP53* and/or *RB1* mutations with concurrent *NOTCH* family mutations correlated with older patient populations, higher tumor grades, and a greater propensity for tumor recurrence [11]. 

Notch signaling has been shown to act as a molecular switch in the skin, promoting epidermal differentiation, and *NOTCH1* ablation results in epidermal hyperproliferation [36,37]. Loss of function in Notch signaling alters sebaceous gland differentiation with a reduction of mature sebocyte density, while conditional *NOTCH1* deletion results in sebaceous gland atrophy [38,39,40]. Mice with pharmacologic inhibition or deficiency of γ-secretase show both disrupted sebaceous gland development and epidermal hyperproliferations with squamous cell carcinomas of the head and neck, respectively, supporting a pleotropic role for Notch in epithelial tissues [41,42].

A potential epigenetic driver of sebaceous carcinoma was observed in the current study, as 4/12 (25%) of all sebaceous carcinomas harbored mutations in *KMT2C*, or lysine methyltransferase 2C, with 2/2 OA tumors harboring missense mutations and 2/2 extraocular tumors harboring deletions. *KMT2C* serves as one of the key regulators of H3K4 methylation, and inactivation results in the development of ureteral neoplasms in mice. It has been reported in breast tumors, glioblastomas, melanoma, and pancreatic cancer in human patients [43,44,45]. Also referred to as *MLL3*, *KMT2C* is a component of the activating signal cointegrator complex, with overexpression documented in a number of epithelial neoplasms [44,46,47]. Mutations in *KMT2D* (*MLL2)*, a closely related methyltransferase, have been identified in squamous cell carcinomas and reported in sebaceous carcinomas with a UV damage mutational signature [33,48]. In another study, an extraocular sebaceous carcinoma harbored a mutation in *MLL3,* while one primary and one metastatic OA harbored mutations in *MLL2* [24].

Previous studies have utilized sequencing approaches to better characterize molecular drivers in sebaceous carcinoma, with one reporting a low incidence of tumors (12/32) harboring more than 5 CNV events, and the most common event resulting from a single copy loss of chromosome 17p, where *TP53* is located [24,33,35]. The current study did not identify high level copy gains in any tumor, consistent with previous findings; however, 54% of tumors harbored equivocal copy gains, and 18% demonstrated copy gains suggestive of amplification in *MYC* [33]. Expression of *MYC* in the basal layers of the epidermis and the proliferative zone at the base of hair follicles has been reported [49,50]. Additionally, growth factor-induced *MYC* promoter activity has been demonstrated in proliferating keratinocytes, while *MYC* knockdown inhibits this proliferation [51,52]. Despite a clear oncogenic role, *MYC* can also stimulate both terminal differentiation of keratinocytes and sebocytes, and in overexpressing transgenic models, induce division of differentiated cells, leading to benign or pre-malignant proliferation [49,53,54,55,56]. 

Additional studies have demonstrated sebaceous gland hyperplasia in *Blimp1*-ablated mice, mediated by loss of *c-myc* repression, and decreased sebocyte size and density with *c-myc* inhibition in *Blimp1+-* induced sebaceous gland organoids [57,58,59]. With respect to epithelial neoplasms, *MYC* amplification has been reported in approximately 50% of squamous cell carcinomas from immunosuppressed patients following organ transplantation, and diffuse nuclear MYC immunolabeling has been demonstrated in eyelid sebaceous carcinomas [60,61]. Additionally, overexpressed *MYC* targets of miRNAs have been reported in sebaceous carcinomas with pagetoid features [22]. 

Due to the relative frequency of *MYC* copy gains in this study, and the well-documented regulatory role for *MYC* in epithelial and sebaceous development, additional evaluation of tumors by FISH and immunohistochemistry was pursued to confirm copy number gains and characterize the potential contribution of *MYC* to sebaceous oncogenesis. FISH identified 4–5 or more signals at 8q24 per nucleus in 70–76% of tumor cells in the two samples with copy gains suggestive of *MYC* amplification by NGS, confirming unequivocal amplification [62]. Similar to previous studies, immunohistochemical analysis of sebaceous carcinomas demonstrated increased MYC labeling of nuclei within intraepithelial and subepithelial tumor cells compared to absent to weak labeling of adjacent normal basilar epidermal, conjunctival, and sebaceous cells [61]. However, there was no correlation between MYC protein expression as measured by h score and mean *MYC* mean log_2_ copy ratio, suggesting that gain of copy does not consistently yield increased levels of MYC protein, or that other mechanisms are driving increased MYC expression. Overall, however, the neoplastic epithelial immunolabeling supports a role for *MYC* in the formation and malignant progression of these tumors. 

Ocular adnexal sebaceous carcinomas, arising from Meibomian glands, glands of Zeis, and sebaceous glands of eyelid skin tend toward a poorly differentiated and more aggressive phenotype. They are reported to be more common in the upper lid where there is a higher density of Meibomian glands [23,63]. The histomorphologic features of sebaceous glands from tamoxifen-inducible *cMYC* mice are well documented, and a few studies have identified *MYC* as a differentially expressed transcription factor and potential therapeutic target in Meibomian gland dysfunction [64,65,66,67,68,69,70]. However, few studies interrogating MYC expression in normal Meibomian glands have been published. To further evaluate MYC expression in the normal Meibomian gland, we pursued characterization in two mouse strains: CD1 and C57B6. In contrast to the reported paucity of nuclear staining in normal eyelid sebaceous glands in humans, strong immunolabeling of MYC was observed in the proliferating cells of the outer layer of the Meibomian gland in both mouse strains [61]. Interestingly, MYC expression in the basal cells of the epidermis and sebaceous glands in these two mouse strains is consistent with previous immunolabeling studies in human skin [49,50].

In summary, our data support the importance of p53 and Rb in sebaceous carcinoma, and also identify a number of other potential oncogenic drivers, including copy number gains at the MYC locus. Our study is limited by the relatively low number of cases successfully sequenced, and the fact that only two extraocular tumors were examined limits statistical comparisons with OA tumors. Additionally, low tumor cellularity also likely contributed to the underestimation of copy gains of the MYC locus in these cases. Nevertheless, further evaluation of MYC gains in tumors, as well as its role in the developing Meibomian gland, may support its role in sebaceous neoplasia [71]. In addition. immunohistochemical or molecular analyses of p53, Rb, Notch1, and MLL3 or its downstream target, H3K4, could also advance our understanding of their pathogenicity in OA sebaceous carcinoma. Future multi-center studies may provide larger numbers of samples and allow more statistical analyses of NGS and other molecular data, as well as additional clinical correlation.

## 4. Materials and Methods

### 4.1. Study Design

Study approval was obtained from the Internal Review Board at our institution. We performed a retrospective search through the Surgical Pathology and Ophthalmic Pathology Archives of The Johns Hopkins Hospital for cases of both OA and extraocular sebaceous carcinoma between 2003–2021. The electronic medical record was reviewed for clinical and pathologic information: Age at presentation, gender, ethnicity, history of malignancy, tumor location, intraepithelial involvement, tumor size, and TNM staging. All cases were reviewed to confirm the diagnosis and determine the amount of tissue remaining. Staging was performed according to the American Joint Committee on Cancer (AJCC) TNM staging system for eyelid carcinoma, 8th edition guidelines.

### 4.2. DNA Extraction and Next-Generation Sequencing

Manual microdissection of FFPE tissue sections followed by DNA extraction and next-generation sequencing (NGS) was performed in the Johns Hopkins Molecular Diagnostic Laboratory using standard clinical protocols and the UCSC version hg19 (NCBI build GRCh37) human reference sequence genome assembly. The Solid Tumor Panel used 435 cancer-related genes, and a complete list can be found in (https://pathology.jhu.edu/jhml-services/assets/test-directory/SolidTumorPanel-II_GeneList_v5.0.pdf; accessed 1 December 2020). Sequences were examined for point mutations and small insertion/deletion mutations in all 435 loci, while for 64 of these copy number variations (CNVs) were also reported. 

Thirteen sebaceous carcinomas were subject to NGS—with all, but Case 2 (12/13), meeting laboratory quality control thresholds. In one sample (Case 11) DNA concentration was sufficient only for a smaller clinical panel of 27 cancer genes (see Supplementary Materials). 

### 4.3. MYC Fluorescence In Situ Hybridization 

Fluorescence in situ hybridization for the *MYC* 8q24.21 rearrangement was performed on FFPE sections of select OA sebaceous carcinomas (*n* = 2) by the Johns Hopkins Cytogenetics Laboratory using standard CLIA approved clinical protocols. The threshold for low level copy number gains suggestive of *MYC* amplification was 5 signals per nuclei. 

### 4.4. MYC Immunohistochemistry

Immunolabeling of FFPE sections of all specimens utilized for NGS was performed by the Department of Pathology clinical laboratory using 5μm sections with a recombinant anti-c-Myc antibody (Abcam ab32072, clone Y69, dilution 1:400; Cambridge, UK) using the Ventana benchmark Ultra platform, ultraView kit (Tucson, AZ, USA), and standard protocols. In human studies, high MYC lymphoma was used as a positive control, while primary antibody was removed in negative controls. Specimens were scored as having no (0), weak (1), moderate (2), or strong (3) immunoreactivity, and the percentage of tumor cells staining was recorded. An h-score was calculated by multiplying the staining intensity score by the percentage (by decile) of positively-labeled tumor cells (or non-neoplastic sebaceous gland). Low h-scores were designated 0–99, and an h score of 100–300 was designated as high. Immunohistochemical scoring was performed independently by two pathologists (CP and CGE). 

Eyelids from normal adult cadaveric C57B6 and CD1 mice (Charles River, Wilmington, MA, USA) were dissected and fixed in 10% NBF for 72 h at room temperature. Samples were processed, paraffin-embedded, sectioned, and stained with hematoxylin and eosin (H&E) by the JHMI Reference Histology Laboratory using standard protocols. Immunohistochemical analysis was performed on tissue sections using MYC (rabbit monoclonal, clone EP121, Epitomics, 1:600; St. Louis, MO, USA) as previously described [72]. In the murine studies, normal prostatic glandular epithelium and prostatic adenocarcinoma were used as negative and positive controls.

### 4.5. Statistical Analyses

Correlation of MYC mean log_2_ ratio with clinicopathologic features, including gender, age at presentation, tumor location, and laterality, MYC h score, and concomitant somatic mutations, was analyzed by Fisher’s exact test and two-tailed *t*-tests. H scores for neoplastic and non-neoplastic sebaceous glands were evaluated using a Student’s t-test. All statistical analyses were performed using Prism 8 GraphPad (v. 9.2.0; San Diego, CA, USA) (α = 0.05).

## 5. Conclusions

We have demonstrated equivocal copy gains of *MYC* in 54% and low-level gains suggestive of amplification in 18% of sebaceous carcinomas, with the latter confirmed by FISH. Further work is necessary to elucidate the role of *MYC* in sebaceous oncogenesis; however, these results indicate it may present a target for precision therapy. A high frequency of mutations in tumor suppressors, including *TP53* and *RB1*, with concurrent *NOTCH1* computations were reported here in addition to both missense mutations and deletions in *KMT2C* in OA and extraocular sebaceous carcinomas, respectively. Identifying genetic and epigenetic mutations and resulting dysregulated cellular pathways as potential targets for personalized treatment of aggressive OA sebaceous carcinomas could facilitate early and more definitive clinical management, prolonging survival and minimizing the need for radical surgical dissections. 

## Figures and Tables

**Figure 3:**
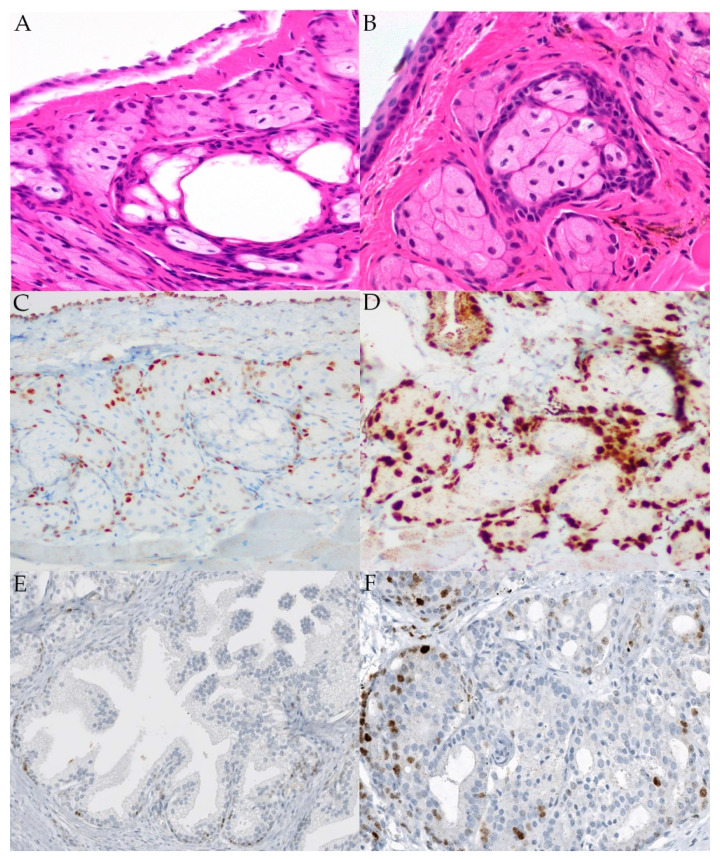
H&E staining and MYC immunolabeling of CD1 (**A**,**C**) and C57B6 (**B**,**D**) murine eyelid. Controls included normal prostatic glandular epithelium (**E**) and prostatic adenocarcinoma (**F**).

**Table 1:** Clinicopathologic characteristics of individual cases (OA, ocular adnexal, EO, extraocular)t).

Case Number	Sex	Age (years)	Tumor Type	Tumor Location	Tumor Laterality	Epithelial Involvement	T Stage	History of Malignancy
1	F	54	OA	Upper lid	Left	None	T1	None
2	M	77	OA	Upper lid	Right	Intraepithelial	T1	None
3	M	83	OA	Upper lid	Left	Combined	T1	Non-cutaneous
4	F	80	OA	Upper lid	Right	Combined	T1	None
5	M	87	OA	Upper lid	Left	Combined	T1	None
6	F	60	OA	Upper lid	Right	Combined	T1	None
7	F	82	OA	Upper lid	Left	Combined	T2	Cutaneous
8	M	83	OA	Upper lid	Right	Subepithelial	T1	Non-cutaneous
9	F	90	OA	Upper lid	Right	Subepithelial	T2	None
10	F	55	OA	Lower lid	Right	Combined	T1	Cutaneous
11	M	43	OA	Upper lid	Right	None	T1	Non-cutaneous
12	F	58	EO	Lateral breast	Left	None	T1	Multiple ^1^
13	M	87	EO	Post-auricular neck	Left	None	T1	Cutaneous

^1^ MTS patient.

**Table 2:** NGS of sebaceous carcinoma (OA: ocular adnexal, EO: extraocular, NE: not evaluated, mutations highlighted in gray do not hold COSMIC annotations).

Case Number	1	2	3	4	5	6	7	8	9	10	11	12	13
Mutations in Clinically Actionable Genes													
*ABL1*									p.E197K				
*ATM*			p.Q2433Pfs*11		p.I2669Yfs*6								
*BRCA1*		p.K1487I								p.R1726G	NE		
*BRCA2*		p.L2510_Y2511insKTCN									NE		
*EGFR*				p.P772S									p.Q976Pfs*9
*FGFR2*												p.S702L	
*FGFR3*											p.F384L		p.R728W
*HRAS*												p.P167Rfs*50, p.P167R	p.P167Rfs*50
*IDH2*									p.V335I				
*MLH1*												p.E694X	
*MSH2*											NE		p.Q409Rfs*4, p.Q409R, p.Q409H, p.I679T
*MSH6*		p.V526L									NE		
*MTOR*		p.L93Qfs*28							p.P677S		NE		
*NF1*		p.K1915T									NE	p.I679Dfs*20, p.C1924Wfs*3	
*NTRK1*			p.H467Q								NE		
*NTRK3*		p.R169C					p.Y604H				NE		
*PALB2*	p.S417Y	p.R1117Sfs*8									NE		
*PDGFRA*		p.E241X											
*PIK3CA*			p.V101fs*0										
*PMS2*		p.V397I			p.S418F	p.L236Sfs*3			p.L236delinsYLLKKIM		NE		
*POLD1*											NE		p.A242T
*PTCH2*											NE	p.G1023S, p.E48del	
*PTEN*								p.P281A					
*RB1*		p.? (Unknown)	p.? (Unknown)		p.Q354Efs*5	p.W75X, p.R358X		p.? (Unknown)	p.E30X	p.R500I			
*RET*												p.A349V	
*ROS1*		p.F1300L		p.D2344N	p.L2337F						NE		
*SMO*										p.L23_G24insL			
*TERT*						p.A279T					NE		
*TP53*		p.R273C	p.G266R	p.? (Unknown)	p.C277F	p.R273C	p.G245S	p.L257P	p.? (Unknown)	p.E339X	p.R196*		
*TSC1*					p.G274S						NE		
*TSC2*		p.G440S									NE		
Genes with Frequent Mutations (≥ 25%)													
												
												
*KMT2C*			p.A1685S				p.S888T				NE	p.K2797Rfs*25	p.K2797Rfs*25
*MNX1*	p.A134_G135insAA	p.A174del	p.A134_G135insAA	p.A134_G135insAA			p.A134_G135insAA		p.A134_G135insAA		NE		
*NOTCH1*								p.G1320Afs*124		p.Y550fs*0	NE	p.R203C	p.L1531Cfs*48, p.P1443Afs*35
*PCLO*		p.G1750E	p.P2128Q								NE	p.E2925D	
*PTPRT*								p.R1209Q	p.R260W		NE	p.P1094Rfs*5	
*MYC* Gain of Copy													
												
												
Mean log_2_ Ratio	<0.5	NE	<0.5	0.833	0.8959	0.5483	0.8178	1.3315	1.4416	<0.5	NE	0.7733	0.6051

**Table 3:** MYC CNV status in sebaceous carcinoma and its relationship to clinicopathologic features (OA: ocular adnexal).

Variable	All Cases (*n* = 11) *n* (%)	No *MYC* Copy Gain (*n* = 3) *n* (%)	*MYC* Copy Gain (*n* = 8) *n* (%)	*p* Value
**Sex**				
Male Female	4 (36) 7 (64)	1 (33) 2 (67)	3 (37) 5 (63)	1.00
**Age (years)**, mean ± SD	73 ± 13.8	64 ± 16.4	76 ± 12.3	0.53
**Tumor site** Ocular adnexal Extraocular	9 (82) 2 (18)	3 (100) 0 (0)	6 (75) 2 (25)	1.00
**Tumor location (OA: *n* = 9)** Upper lid Lower lid	7 (78) 2 (22)	2 (67) 1 (33)	5 (83) 1 (17)	1.00
**Tumor laterality (OA: n = 9)** Right Left	5 (56) 4 (44)	1 (33) 2 (67)	4 (67) 2 (33)	0.52

**Table 4:** MYC CNV status in sebaceous carcinoma and its relationship to MYC protein expression, tumor size, and tumor suppressor mutations.

Variable	All Cases (*n* = 11) *n* (%)	No *MYC* Copy Gain (*n* = 3) *n* (%)	*MYC* Copy Gain (*n* = 8) *n* (%)	*p* Value
**MYC expression (IHC)**				
Low High	5 (45) 6 (55)	1 (33) 2 (67)	4 (50) 4 (50)	1.00
**Tumor size (mm)**, mean ± SD	7.7 ± 2.6	6.5 ± 0.7	8.0 ± 2.9	0.50
***TP53*** Mutation WT	8 (73) 3 (27)	2 (67) 1 (33)	6 (75) 2 (25)	1.00
***RB1*** Mutation WT	6 (55) 5 (45)	2 (67) 1 (33)	4 (50) 4 (50)	1.00

MYC expression was also evaluated in two common laboratory strains of mice. In both strains, MYC was localized to the basilar epithelial layers of the Meibomian glands, as shown in Figure 3.

## Supplementary Materials

The following are available online at https://www.mdpi.com/article/10.3390/ijms22168454/s1, Figure S1: Frequency of genes with equivocal copy number gains. NGS Solid Tumor Panel-Limited Methodology for Case 11.
