# Supplementary material for: NGS Analysis Confirms Common TP53 and RB1 Mutations, and Suggests MYC Amplification in Ocular Adnexal Sebaceous Carcinomas
Source: Int J Mol Sci. 2021 Aug 6;22(16):8454. doi: 10.3390/ijms22168454 (PMC8395148; doi:10.3390/ijms22168454)
Supplement: Supplementary file 1 [file ijms-22-08454-s001.zip › Supplemental Figure S1.pdf]

Supplementary Materials:

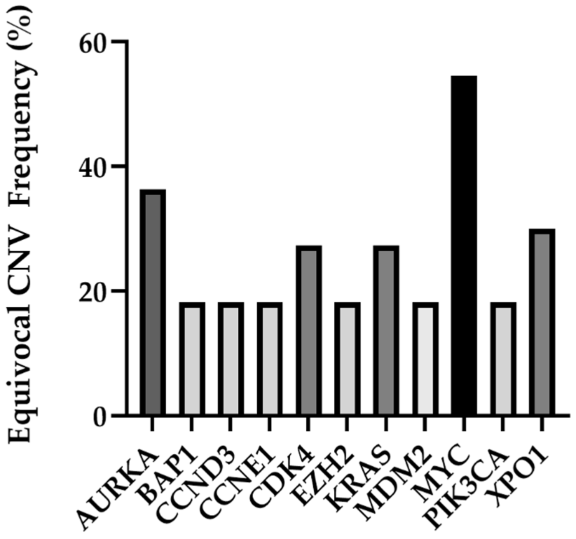

Supplemental Figure S1. Frequency of genes with equivocal copy number gains (mean $\log_2$ ratio > 0.5) in sebaceous carcinomas.

NGS Solid Tumor Panel-Limited Methodology (Case 11 only):

This assay uses next-generation sequencing to analyze the most frequently mutated regions of the following 27 cancer genes: *ABL1* (NM_007313), *AKT1* (NM_005163) , *ATM* (NM_000051) , *BRAF* (NM_004333) , *CDKN2A* (NM_000077), *EGFR* (NM_005228), *ERBB2* (NM_004448), *FGFR1* (NM_015850), *FGFR2* (NM_000141), *FGFR3* (NM_000142), *GNA11* (NM_002067), *GNAQ* (NM_002072), *HRAS* (NM_005343), *IDH1* (NM_005896), *IDH2* (NM_002168), *KIT* (NM_000222) , *KRAS* (NM_004985), *MET* (NM_000245), *MLH1* (NM_000249), *NRAS* (NM_002524), *POGFRA* (NM_006206), *PIK3CA* (NM_006218), *PTEN* (NM_000314), *RB1* (NM_000321) , *RET* (NM_020975), *SMO* (NM_005631), and *TP53* (NM_000546).
